# A machine learning method for improving the accuracy of radiation biodosimetry by combining data from the dicentric chromosomes and micronucleus assays

**DOI:** 10.1038/s41598-022-25453-2

**Published:** 2022-12-06

**Authors:** Igor Shuryak, Ekaterina Royba, Mikhail Repin, Helen C. Turner, Guy Garty, Naresh Deoli, David J. Brenner

**Affiliations:** 1 Center for Radiological Research, Columbia University Irving Medical Center, 630 West 168th Street, VC-11-234/5, New York, NY 10032 USA; 2 Radiological Research Accelerator Facility, Columbia University Irving Medical Center, Irvington, NY USA

**Keywords:** Predictive markers, Machine learning, Assay systems

## Abstract

A large-scale malicious or accidental radiological event can expose vast numbers of people to ionizing radiation. The dicentric chromosome (DCA) and cytokinesis-block micronucleus (CBMN) assays are well-established biodosimetry methods for estimating individual absorbed doses after radiation exposure. Here we used machine learning (ML) to test the hypothesis that combining automated DCA and CBMN assays will improve dose reconstruction accuracy, compared with using either cytogenetic assay alone. We analyzed 1349 blood sample aliquots from 155 donors of different ages (3–69 years) and sexes (49.1% males), ex vivo irradiated with 0–8 Gy at dose rates from 0.08 Gy/day to ≥ 600 Gy/s. We compared the performances of several state-of-the-art ensemble ML methods and found that random forest generated the best results, with R^2^ for actual vs. reconstructed doses on a testing data subset = 0.845, and mean absolute error = 0.628 Gy. The most important predictor variables were CBMN and DCA frequencies, and age. Removing CBMN or DCA data from the model significantly increased squared errors on testing data (p-values 3.4 × 10^–8^ and 1.1 × 10^–6^, respectively). These findings demonstrate the promising potential of combining CBMN and DCA assay data to reconstruct radiation doses in realistic scenarios of heterogeneous populations exposed to a mass-casualty radiological event.

## Introduction

In the current global environment there continues to be the potential of military conflicts, terrorist activities and/or accidents involving large-scale exposures of people to ionizing radiation. The general population is not equipped with physical radiation dosimetry devices, and consequently there is an important need to develop and perfect biodosimetry^1–3^ approaches, which can reliably estimate the radiation dose absorbed by each exposed person, based on samples of biological materials (e.g. blood) from that person. Obtaining such dose reconstructions for the potentially large number of exposed persons in a reasonably short time is important for providing accurate individual-specific information to potentially exposed persons (including the “worried well”), for performing appropriate triage and prescribing treatment regimens if needed, and potentially for predicting long-term health risks.

Among the large and growing variety of currently available radiation biodosimetry techniques, cytogenetic damage endpoints measured by the dicentric chromosome (DCA) and cytokinesis-block micronucleus (CBMN) assays remain the most accurate and reliable options^4^. The DCA assay is the current “gold standard” in biodosimetry^5^. Cytogenetic biodosimetry techniques continue to be improved and developed^6–11^, and implementation of these assays using high-throughput automated approaches is becoming more widespread^12–17^. The automatic scoring technologies are advantageous because they can be employed in mass-casualty situations.

There is also growing recognition of the potential utility of the rapidly evolving field of machine learning (ML) for radiation biodosimetry, particularly in combination with high-throughput automated scoring techniques^18–20^. Such combined approaches have the capacity to rapidly generate individualized radiation dose reconstructions based on data from blood samples obtained from vast numbers of people affected by a large-scale radiological event such as an improvised nuclear device (IND) detonation^2,11,21–26^. This is possible because ML can integrate multiple types of data inputs, such as different biodosimetry assay results as well as other variables including demographic data on the exposed individuals, and use these data to produce predictions (i.e. reconstructions) of the absorbed radiation dose. As an example of this approach, we have recently demonstrated that ML tools are promising for high-throughput biodosimetry in complex exposure scenarios where neutrons are present together with photons^27^.

In the current study, we investigated the capabilities of ML to improve absorbed dose reconstructions by *combining data from automated DCA and CBMN assays*. We hypothesize that using DCA and CBMN output together, in the context of high-throughput automatic scoring systems, will provide superior dose reconstruction accuracy, compared with using either assay alone. The rationale for this hypothesis is that dicentric chromosomes and micronuclei have different radiation dose response shapes and dependences after exposure to different dose rates of ionizing radiation on demographic variables^28,29^, which enables each of these assays to complement each other. In other words, the information provided by the DCA and CBMN assays is not completely redundant, and the contributions of each assay can be exploited by ML to improve the accuracy of dose predictions. For example, CBMN yields can turn over and start to decrease at lower doses than DCA yields, which implies that if DCA is high (did not turn over) but CBMN is low (already turned over) in a given sample, then the sample could have received a high radiation dose.

This hypothesis is novel for the following reason: Although ML approaches were previously used for biodosimetry-related image analysis tasks, we consider that our group at the Columbia University Irving Medical Center (CUIMC) is perhaps the first to implement ML directly for dose reconstruction, using data from automated scoring systems. To our knowledge, dose reconstruction using *both* DCA and CBMN data as predictor variables within a single ML model (instead of using each assay *alone*) was never previously implemented.

Such a combined biodosimetry strategy could be particularly useful in realistic scenarios for mass-casualty events, where: (1) The population of potentially irradiated individuals is very heterogeneous in terms of age, sex and other factors. (2) Radiation can be delivered at very different dose rates, e.g. extremely high dose rate “prompt” exposures within the first fraction of a second after a nuclear device detonation^30^, followed by protracted exposures from radioactive fallout days-weeks later^31^. To mimic such scenarios in the laboratory, we collected and analyzed blood samples from 155 donors of different ages (3–69 years) and sexes (49.1% males), ex vivo irradiated with 0–8 Gy of photon or electron beams at dose rates varying from 0.08 Gy/day to > 600 Gy/s. Both DCA and CBMN assays were performed on aliquots taken from each blood sample, and analyzed in the same laboratory. The potential advantages of this approach include the following aspects: (1) For these two assays one biological material is used—peripheral blood. (2) Both assays are cytogenetic, and can thus be performed on the same equipment and using the same reagents, such as culture medium, hypotonic solution, fixative and DNA dye (DAPI), during some of the assay steps. (3) The proposed approach described here can be introduced in different cytogenetic biodosimetry laboratories that use both of these assays.

The current study represents a proof of principle for the innovative concept of combining data from several automated radiation biodosimetry assays to improve radiation dose reconstructions. Presented here, we used DCA and CBMN assays, but the concept of combining multiple predictor variables in ML-based biodosimetry is potentially extendable to other types of radiation-induced damage biomarkers. Combining data from multiple assays has the potential of increasing the reliability of dose reconstructions, and possibly overcoming confounders, which may not affect all assays equally.

## Materials and methods

### Experimental procedures

Our group’s methodology for ex vivo irradiation of human donor blood samples and implementation of automated DCA and CBMN assays on these samples is described in detail elsewhere^8–10,32^. It is summarized briefly below.

This study was approved by Columbia University’s Institutional Review Board (IRB) protocol IRB-AAAF2671. Blood from pediatric donors (Sterling IRB protocol #8933) was collected by Jean Brown Clinical Research (Salt Lake City, UT) into sodium heparin vacutainer tubes, and shipped overnight in a temperature-controlled shipper (22 °C Credo Cube, Fisher Scientific, Pittsburgh, PA). Blood from adults was collected at Columbia University Irving Medical Center. Blood was then aliquoted into 2D-barcoded tubes (Matrix Storage Tubes, Thermo Fisher). All recruited blood donors filled in a questionnaire, which included questions about exposure to X rays, CT scans, chemotherapy, or radiotherapy within the last 6 months. Potential donors with such exposures were excluded from the study. All methods were performed in accordance with the relevant guidelines and regulations. Informed consent was obtained from all subjects and/or their legal guardian(s).

High dose rate irradiations were performed using a custom-modified Varian Clinac irradiator^30^. Blood sample aliquots from different donors were irradiated using 6 or 9 MeV electron pulses. Pulse durations were between 0.1 and 4 µs. Detailed dosimetry was performed using EBT-3 film and/or a NIST-traceable Advanced Markus Ion Chamber^30^.

Low dose rate irradiations that simulate radioactive fallout after a nuclear explosion were performed using a combination of our modified XRAD 320 machine^33^ and the VADER^31^, which delivers a photon dose rate of 0.1–1.0 Gy/day. Blood sample aliquots were housed in a customized plastic incubator^33^, which was placed into the irradiation chamber. Dosimetry was performed daily using a calibrated 10 × 6–6 ion chamber (Radcal, Monrovia, CA).

Before irradiations, we dilute whole blood in RPMI in 1:4 ratio. After that, we transfer 150 µl (30 µl blood + 120 µl RPMI) of diluted blood into plates, centrifuge it and replace 120 µl of old RPMI with 270 µl of fresh PB-max. After each type of irradiation, the diluted blood aliquots were transferred into 96 well plates, and processed for DCA and CBMN assays using the RABiT-II automatic scoring system^8–10^. Imaging-based identification of dicentric chromosomes or micronuclei was implemented using a BioTek Cytation Cell Imaging Multi-Mode Reader (with 20× objective) and analyzed using a custom software, FluorQuant v.6.1 (micronucleus assay) and FluorQuantDic v.4 (dicentric assay), written in Visual C++ using the OpenCV computer vision libraries (Version 3.1, http://www.opencv.org).

### Data set

The main variables in the resulting data set were: The yield of dicentric chromosomes per monocentric chromosome (Yield). The yield of micronuclei per binucleated cell (Mi_BN). Sex, coded as a binary variable with 0 = males and 1 = females. Race, coded as a categorical variable with 0 = unreported; 1 = African American; 2 = Asian; 3 = White; 4 = Mixed. Ethnicity, coded as a categorical variable with 0 = unreported; 1 = Hispanic/Latino; 2 = non-Hispanic/Latino. The radiation dose (Dose, in Gy), which was eventually treated as the target variable to be predicted by the ML model using the variables listed above as predictors. The data set consisted of 1349 blood sample aliquots from 155 donors^32^.

The radiation dose rate was converted into ordinal categories (Dose_rate_category) as follows: 0 = very low dose rate (approximately 0.08 Gy/day, with dose delivered over 48 h) using our custom-built VADER irradiator^31^; 1 = 1 Gy/min; 2 = 1 Gy/s; 3 = approximately 600 Gy/s (3 Gy in 2 electron pulses or 8 Gy in 3 pulses with 5.6 ms between pulses); 4 = single 5 µsec electron pulse. By default, we did not include Dose_rate_category in the set of predictor variables to train ML algorithms for dose reconstruction because: (1) dose rate information would not be available in a realistic mass casualty biodosimetry scenario; (2) we were interested to investigate whether or not the proposed approach of combining DCA with CBMN data by ML would be able to decently reconstruct radiation doses even when dose rate is unknown and can vary over a very wide range. However, to assess the potential effect of dose rate on ML model predictions, we also fitted a model version where Dose_rate_category was included in the predictor set. In this case, unexposed samples (with Dose = 0 Gy) were randomly assigned to any of the dose rate categories from 0 to 4.

### Data pre-processing

The data set, composed of DCA and CBMN data for each aliquot of blood from each donor for each irradiation condition and replicate, was imported for analysis using the *R* 4.2.0^34^ programming language. Blood sample aliquots with < 20 binucleated cells (BN) or < 20 monocentric chromosomes (MC) were removed because these samples were likely to produce unreliable DCA or CBMN data due to low numbers of counted events. The number of retained samples in this data set was 1122, provided in Supplementary_File_1 online. Summary statistics for these samples, split into training and testing halves, are shown in Table 1. Among the 1122 samples retained for analysis, 145 received 0 Gy, 541 received 3 Gy, 6 received 4 Gy, and 430 received 8 Gy. The initial number of samples was 1349, so approximately 17% of the samples were excluded from analysis.

**Table 1:** Summary statistics for the training and testing halves of the analyzed data set.

Training half of data set	Dose	Age	Race 1	Race 2	Race 3	Ethnicity 1	Ethnicity 2	Yield	Mi_BN	Mi_BN_c
Mean	4.520	24.107	0.064	0.171	0.624	0.250	0.365	0.054	0.216	0.647
Std	2.881	16.511	0.245	0.377	0.485	0.433	0.482	0.038	0.183	0.369
Min	0	3	0	0	0	0	0	0.004	0.000	0.083
25%	3	11	0	0	0	0	0	0.026	0.067	0.411
50%	3	22	0	0	1	0	0	0.044	0.159	0.537
75%	8	36	0	0	1	0	1	0.071	0.317	0.832
Max	8	69	1	1	1	1	1	0.213	0.905	2.043

Testing half of data set	Dose	Age	Race 1	Race 2	Race 3	Ethnicity 1	Ethnicity 2	Yield	Mi_BN	Mi_BN_c
Mean	4.547	22.289	0.068	0.148	0.649	0.225	0.389	0.053	0.215	0.652
Std	2.919	15.638	0.252	0.355	0.478	0.418	0.488	0.038	0.184	0.382
Min	0	3	0	0	0	0	0	0.000	0.003	0.078
25%	3	9	0	0	0	0	0	0.024	0.065	0.411
50%	3	21	0	0	1	0	0	0.042	0.161	0.548
75%	8	29	0	0	1	0	1	0.076	0.320	0.805
Max	8	69	1	1	1	1	1	0.219	0.950	1.995

There were 561 samples in each half. As described in the main text, Dose is the radiation dose (Gy) to which each sample was exposed, Age is the age of the subject (years), Yield is the dicentrics yield, Mi_BN and Mi_BN_c are the raw and corrected micronuclei indeces. Race and Ethnicity are categorical variables, so they were converted into columns of binary variables by one-hot encoding.

Since the raw micronucleus yield per binucleated cell (Mi_BN) decreased at high radiation doses (at 8 Gy compared with 3 Gy), we created a corrected “linearized” micronucleus index (Mi_BN_c). It was calculated as follows, where Mi is the number of micronuclei in the sample, BN is the number of binucleated cells, MN is the number of mononucleated cells, and *k* is an adjustable model parameter:

1 $$Mi\_BN\_c=\frac{Mi}{BN}+ \left (\frac{1}{k}\right)\frac{MN}{BN}$$

We used quantile regression (*quantreg R* package implementation) to model the dose response of the median (50th percentile) of Mi_BN_c using a linear quadratic (LQ) function. The parameters of this function are α and β, and a baseline value (intercept) *c*, described by the following equation, where *D* is dose (in Gy):

2 $$Median\left(Mi\_BN\_c\right)=c+\alpha D+\beta {D}^{2}$$

During the fitting procedure for Eqs. (1, 2), we varied parameter *k* in Eq. (1) in increments, so that the β term in Eq. (2) approached zero and became statistically consistent with zero. Therefore, parameter *k* was incrementally adjusted (in steps of 10 units) such that the median of the resulting Mi_BN_c index would approach a linear dose response over the studied dose range of 0–8 Gy. This goal was achieved by *k* = 70. The resulting Mi_BN_c index, which represents an additional engineered feature for dose reconstruction, was added to the data set provided in Supplementary_File_1 online.

### Machine learning analyses

**Figure 1:**
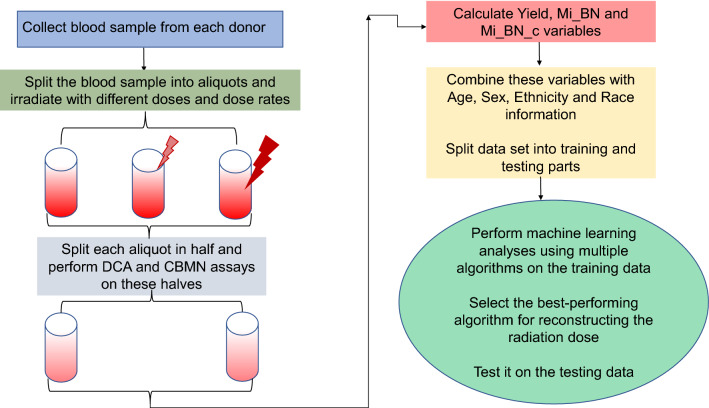
Schematic representation of the experimental design for this study. Details are explained in the main text.

Our main goal in this study was to implement ML-based regression approaches to estimate (reconstruct) the radiation dose received by each blood sample. A schematic representation of the experimental design for this study is provided in Fig. 1.

We used the Boruta feature selection algorithm (implemented by the *Boruta R* package)^35^ to identify and discard any weak predictor variables, which would not be useful for reconstructing dose in this data set. Boruta iteratively compares the importance score of each predictor with the importance score of its randomly shuffled “shadow”, in the context of a random forest model^36^. It duplicates the data set and randomly shuffles the values in each column. These shuffled values are called shadow features, and they are re-created in each iteration. Those predictors that had significantly (p-value < 0.05 with Bonferroni correction) worse importance scores than the shadow features during Boruta implementation on a randomly selected training half of the data were discarded from further analysis.

We considered several state of the art tree ensemble ML methods as useful approaches for the task of dose reconstruction on the data set containing the retained predictors. Tree ensembles such as random forest (RF)^36^, XGBoost^37^, LightGBM^38^, and CatBoost^39^ represent a popular group of ML algorithms, which tend to perform well on data sets composed of tabular data, such as the one analyzed here. Such methods fit many models (decision trees) and combine them into an ensemble, which tends to produce more reliable predictions than a single model. Their strengths include the ability to model non-linear relationships and interactions between variables, and low sensitivity to correlations between predictor variables and to outlier observations.

The RF algorithm, pioneered by the famous American statistician Leo Breiman^36^, generates many uncorrelated decision trees by bootstrap aggregation, or “bagging” (randomly selecting samples from training data with replacement) and feature randomness (selecting a random subset of predictor variables for each tree). Predictions from all trees are then averaged for regression problems such as the one here.

By comparison, the boosting strategy uses an iterative approach where trees are added to the ensemble sequentially, so that each next tree attempts to improve the fit to those data instances, which were poorly fitted by previous trees. State of the art boosting algorithms include XGBoost, LightGBM, and CatBoost. XGBoost was created at the University of Washington in USA and became widely popularized due to its strong performance, for example in various ML competitions^37^. LightGBM, developed by the Microsoft corporation, differs by using the Gradient-Based One-Sided Sampling (GOSS) technique, which updates a given tree using a selection of the largest gradients and randomly sampled small gradients^38^. CatBoost was developed by the Yandex company in Russia, and is optimized for handling categorical variables automatically, with no need for manual pre-processing (such as one hot encoding) by the user^39^.

We implemented these ML algorithms in the Python 3.10.5 programming language, using the Jupyter notebooks interface (https://jupyter.org/). To establish some “baseline” of performance for comparison with the algorithms listed above, we used several other algorithms: linear regression, elastic net regression, support vector machines regression (SVR), and the linear-tree package (https://github.com/cerlymarco/linear-tree) which builds trees with linear models at the leaves. Linear regression was used because it can be regarded as the “simplest” type of modeling tool for the data analyzed here. Elastic net is a regularized regression method which implements both L1 and L2 penalties, often resulting in improved performance compared with linear regression, and/or with regularized algorithms which use one of these penalties but not both^40^. SVR is an adaptation of the powerful support vector machines ML algorithm, which uses a “geometric” approach to separate data classes, from classification to regression problems^41^. All three methods were implemented using sklearn in Python: the LinearRegression, ElasticNetCV and SVR packages, respectively. We expected all of these algorithms to perform somewhat worse than the more flexible RF and boosting methods listed above.

To mitigate the problem of overfitting, which can affect all model types, we trained each ML model using repeated *k*-fold cross validation (fivefold, repeated 30 times) on a randomly selected ¼ of the data, and evaluated each model on another ¼ of the data. The remaining ½ of the data was set aside for ultimate testing (validation) of the preferred model, which was identified using the initial comparison of models.

Three performance metrics were used to evaluate each ML model during the initial model comparison: mean absolute error (MAE), root mean square root error (RMSE) and coefficient of determination (R^2^). These first two metrics are described in Eqs. (3–4) below, where *D* represents the actual dose and $\widehat{D}$ represents predicted (reconstructed) dose, calculated for *i* = 1..N data points.

3 $$\mathrm{MAE }= \frac{1}{N }\sum \limits_{i=1}^{N}|{D}_{i }-\widehat{{D}_{i}}|$$

4 $$\mathrm{RMSE }=\sqrt{\frac{1}{N }\sum \limits_{i=1}^{N}{({D}_{i }- {\widehat{D}}_{i})}^{2}}$$

The last metric (R^2^) is the square of the Pearson correlation coefficient between actual and predicted doses.

We compared all three metrics across the evaluated ML models to select the best-performing preferred model, and the second-place model. Both of those models were refined by hyperparameter tuning in Python and *R* using grid search strategies, and the best tuned versions were evaluated using the same three performance metrics on the originally withheld ½ of the data—the testing set.

We used Shapley Additive exPlanations (SHAP)^42^ to identify which features (predictor variables) had the greatest impact on the dose reconstructions generated by the preferred top two models. The SHAP approach originated in the fields of economics and game theory, but it is also quite useful for interpreting ML models. An important strength of the SHAP methodology is that it estimates the contribution of each feature to the model's predictions, taking into account the multitude of possible orders in which the feature of interest could be added to the model. The SHAP calculation is summarized below, where *F* represents the number of features in the model, *S* represents a subset of these features, *v* is the function that generates the value of the model’s prediction based on the features (the reconstructed dose in this case), *i* is the index of the feature of interest, and SHAP_i_ is the SHAP value of feature *i*:

5 $$\begin{aligned}{SHAP}_{i} & =\sum_{S\subseteq F-i}\left[\frac{\left|S\right|!\left(\left|F\right|-\left|S\right|-1\right)!}{\left|F\right|!}\left(v\left(S{U}_{i}\right)-v\left(S\right)\right)\right] \\ & =\frac{1}{\left|F\right|}\sum_{S\subseteq F-i} \left [{ \left(\begin{array}{c}\left|F\right|-1\\ \left|S\right|\end{array} \right)}^{-1}(v\left(S{U}_{i}\right)-v(S)) \right]\end{aligned}$$

The terms in this equation have the following interpretations. $\frac{1}{\left|F\right|}$ is a scaling factor. $S\subseteq F-i$ indicates that the feature of interest (*i*) is excluded from the set for the current calculation. $${(\begin{array}{c}\left|F\right|-1\\ \left|S\right|\end{array})}^{-1}$$ represents how many groups of size |*S*| can be formed from |*F*|-1 features. $v\left(S{U}_{i}\right)-v(S)$ represents the marginal value of adding feature *i* to the set, i.e. a comparison of the model’s prediction values when feature *i* is included *vs* excluded from the set. We used the shap.Explainer in Python and the *fastshap* and *SHAPforxgboost* packages in *R* to calculate SHAP values for various features in the selected ML models.

## Results

**Figure 2:**
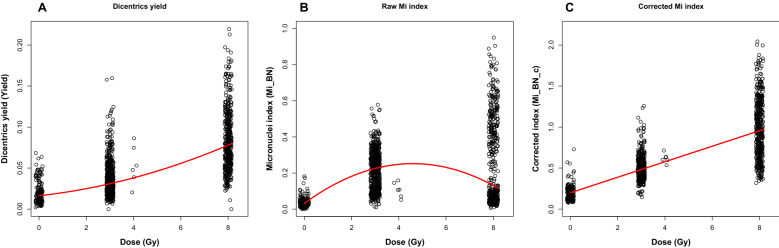
Dose responses for the yields of dicentric chromosomes (per total number of chromosomes in the sample, (**A**), and raw micronuclei (**B**) and corrected (linearized) micronuclei (**C**) per cell. Circles represent the data for individual blood samples, and curves represent linear-quadratic quantile regression fits that describe the median (50th percentile) of the distribution of each variable. The data (circles) were randomly “jittered” by small amounts along the x-axis to improve visualization by reducing overlap. The dose values were 0, 3, 4, and 8 Gy.

Radiation dose responses for the DCA and CBMN assays are shown in Fig. 2. Linear quadratic (LQ) quantile regression for the median of dicentric chromosome yield (Yield, Fig. 2A) produced the following parameters: baseline value in unexposed samples, c = 1.63 × 10^–2^ ± 1.30 × 10^–3^ (standard error), p-value < 10^–6^; linear dose response term α = 3.22 × 10^–3^ ± 8.20 × 10^–4^ Gy^−1^, p-value 1.0 × 10^–4^; quadratic dose response term β = 5.60 × 10^–4^ ± 1.00 × 10^–4^ Gy^−2^, p-value < 10^–6^. For raw micronucleus yield (Mi_BN, Fig. 2B), the regression parameters were: c = 3.19 × 10^–2^ ± 1.56 × 10^–3^, p-value < 10^–6^; α = 9.60 × 10^–2^ ± 5.04 × 10^–3^ Gy^−1^, p-value < 10^–6^; β = -1.05 × 10^–2^ ± 9.50 × 10^–4^ Gy^−2^, p-value < 10^–6^. The decrease in Mi_BN at 8 Gy compared with 3 Gy prompted us to develop the corrected “linearized” micronucleus index Mi_BN_c. Using *k* = 70 in Eq. (1) led to a small and non-significant β term in Eq. (2): 1.60 × 10^–4^ ± 9.60 × 10^–4^ Gy^−2^ (p-value 0.87). Dropping this non-significant β term produced the following parameters for a linear dependence of the median Mi_BN_c (Fig. 2C) on dose: c = 0.200 ± 2.14 × 10^–3^, α = 9.45 × 10^–2^ ± 2.33 × 10^–3^ Gy^−1^.

**Figure 3:**
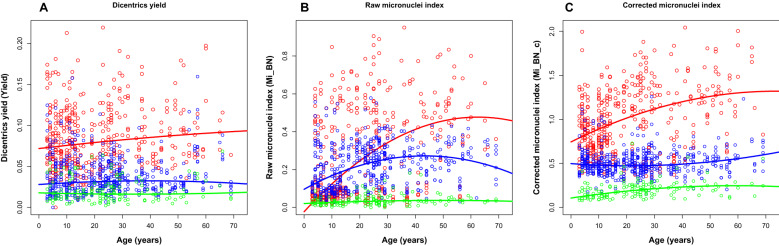
Age dependences for yields of dicentric chromosomes (**A**), and raw micronuclei (**B**) and corrected (linearized) micronuclei (**C**). Green = 0 Gy, blue = 3 Gy, red = 8 Gy. Circles represent the data for individual blood samples, and curves of each corresponding color represent linear-quadratic quantile regression fits that describe the median (50th percentile) of the distribution of each variable.

The dependences of Mi_BN, Mi_BN_c and Yield on age and radiation dose are shown in Fig. 3. The fitted curves represent LQ quantile regressions for the median of each variable at each dose (0, 3 or 8 Gy). These results suggest that the DCA and CBMN assay yields, particularly Mi_BN_c and Yield, tended to increase with age. This age-related increase, especially in baseline values (i.e., at 0 Gy), may reflect reduction in DNA repair efficiency and induction of genomic instability due to aging and factors such as tobacco smoking^43–45^.

The initial feature selection procedure using the Boruta algorithm rejected the Sex variable as a weak predictor, which outperformed random noise in only 35.8% of the iterations. By comparison, Race outperformed noise in 82.8% of iterations, Ethnicity in 98.5%, and all other predictors (Age, Mi_BN, Mi_BN_c and Yield) in 100%. Consequently, Sex was discarded from further analysis. This finding suggests that sex of the blood donors did not have a significant effect on dose reconstructions in this data set, although other research suggests that sex may play a role in cytogenetic assays^28,46^. Specifically, the absence of a sex effect is consistent with the results from manual scoring of dicentrics^47^, but not micronuclei^48^.

Using the retained predictor variables, we compared the performances of several state-of-the-art ML methods, assessing their abilities to reconstruct radiation dose in a heterogeneous population of samples exposed to different dose rates. The results of the initial model comparison (Table 2) suggest that RF and CatBoost had the best and second-best performances, respectively.

**Table 2:** Initial comparison of ML model performances for dose reconstruction.

Model type	R^2^	RMSE (Gy)	MAE (Gy)
Random forest (RF)	0.840	1.123	0.567
CatBoost	0.824	1.176	0.661
LightGBM	0.821	1.186	0.745
XGBoost	0.785	1.300	0.659
LinearRegression	0.732	1.450	1.155
SVR	0.699	1.538	1.112
ElasticNet	0.697	1.544	1.178
LinearBoost	0.687	1.568	1.108

All models were trained on a randomly selected ¼ of the data set, and evaluated using R^2^, RMSE and MAE metrics on another ¼ of the data set. As described in the main text, the remaining ½ of the data was set aside for ultimate testing of the preferred models.

Hyperparameter tuning by grid search approaches was used on these top two ML algorithms—RF and CatBoost. Their performances were evaluated on the testing data set, which was not seen by any of the models during training. RF, tuned in *R* using the *ranger* and *caret* packages, ultimately performed the best on testing data, with R^2^ for actual vs. reconstructed doses = 0.845, RMSE = 1.160 Gy and MAE = 0.628 Gy. Its best hyperparameters were: number of decision trees in the forest, num.trees = 500; the variable importance measure, importance = "permutation"; the rule by which each split in a tree occurs, splitrule = "extratrees"; minimum number of data instances in a terminal node, min.node.size = 1; the number of features considered in each tree, mtry = 6. To assess how RMSE and MAE metrics varied by the actual radiation dose, we calculated them separately for 0, 3 and 8 Gy samples. For 0 Gy, RMSE = 0.954 Gy and MAE = 0.476 Gy. For 3 Gy, the corresponding values were 1.128 and 0.566 Gy, and for 8 Gy they were 1.255 and 0.750 Gy, respectively. As expected, the error metric magnitudes increased somewhat as function of increasing dose, but this tendency was not very dramatic.

**Figure 4:**
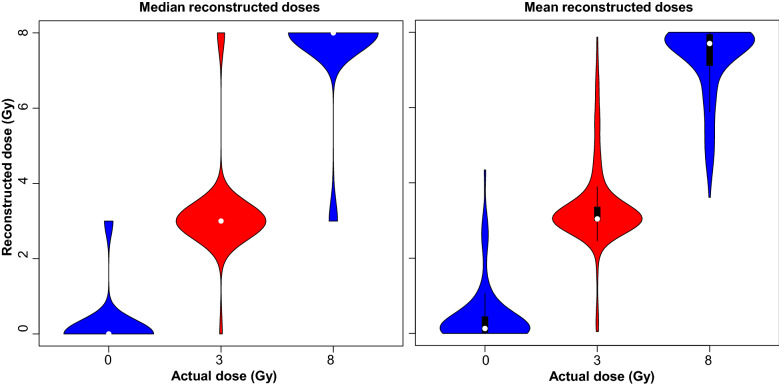
Visualization of actual and reconstructed radiation doses. The reconstruction was performed by the random forest algorithm on the testing half of the data set. The left panel shows median dose predictions, generated using quantile random forest, and the right panel shows mean dose predictions. The violin plots for 0, 3 and 8 Gy show the distributions of corresponding reconstructed dose values. The model’s performance metrics were: R^2^ for actual vs. reconstructed doses = 0.845, RMSE = 1.160 Gy and MAE = 0.628 Gy. The median reconstructed doses which corresponded to actual doses of 0, 3 and 8 Gy were 0.132, 3.043 and 7.700, respectively.

Predictions (dose reconstructions) for the best-performing RF model on testing data are provided in Supplementary_File_2 online and displayed graphically in Fig. 4. In addition to predictions for the mean reconstructed dose, we calculated quantile predictions from an RF model with the same hyperparameters, using the *ranger R* package with the quantreg = TRUE option. The quantiles were 0.05, 0.1, 0.25, 0.5, 0.75, 0.9, 0.95, and corresponding predicted values are labeled as Reconstructed_Dose_q5 to Reconstructed_Dose_q95 in Supplementary_File_2 online.

The mean and median (Reconstructed_Dose_q50) predictions for reconstructed dose are shown in Fig. 4. The small number of samples at a dose of 4 Gy were excluded from the figure to improve visualization, but RF predictions for all testing data samples are provided along with corresponding actual doses in Supplementary_File_2 online (Reconstructed_Dose and Actual_Dose columns). The median for absolute errors was only 0.15 Gy, the 75th percentile was 0.72 Gy, and 1 Gy corresponded to approximately the 80th percentile. In other words, for approximately 80% of testing data points, absolute errors on the radiation doses were ≤ 1 Gy.

Out of the 561 testing data points, 553 actual doses fit within the 0.1 to 0.9 quantiles of RF predictions, and 548 fit within the 0.25 to 0.75 quantiles. In other words, only 13 out of 561 testing data points (about 2.3%) had reconstructed doses outside the 25th to 75th percentile range of the quantile predictions by the RF model, which suggests that the model made large errors in dose reconstruction only infrequently.

CatBoost tuned in Python using the GridSearchCV procedure from sklearn.model_selection also performed decently, but somewhat worse than RF: R^2^ = 0.800, RMSE = 1.304 Gy, MAE = 0.783 Gy. Its best hyperparameters were: the number of trees in the ensemble, iterations = 425; learning rate during the training process, learning_rate = 0.085; L2 regularization of the loss function, l2_leaf_reg = 3; maximum allowed tree depth, depth = 7. The squared errors for CatBoost on testing data were significantly larger than those for RF (p-value 1.89 × 10^–4^ using the scipy.stats.wilcoxon test).

Predictions for the CatBoost model on testing data are displayed graphically in Supplementary Fig. 1. Their distributions look visually similar to those from RF (Fig. 4), but there is a small fraction of predicted dose values outside the range of the training data (i.e. < 0 or > 8 Gy). Since the RF algorithm uses bagging to build the tree ensemble, it cannot extrapolate beyond the range of training data. Boosting algorithms such as CatBoost, however, successively fit trees to the residuals of the fit from the previous step and can stray somewhat out of the training range (from -0.34 to 8.69 Gy in this case). This difference in ensemble building approach may explain why on this data set RF produced somewhat better performance metrics that any of the boosting algorithms, but both strategies may ultimately be useful for biodosimetry under field conditions.

Expectedly, both the RF and CatBoost models performed slightly worse on testing data than during initial training (Table 2). The magnitude of performance decrease between training and testing was not severe for either model, and suggests that both algorithms were able to generalize relatively well from one portion of the data set to another.

**Figure 5:**
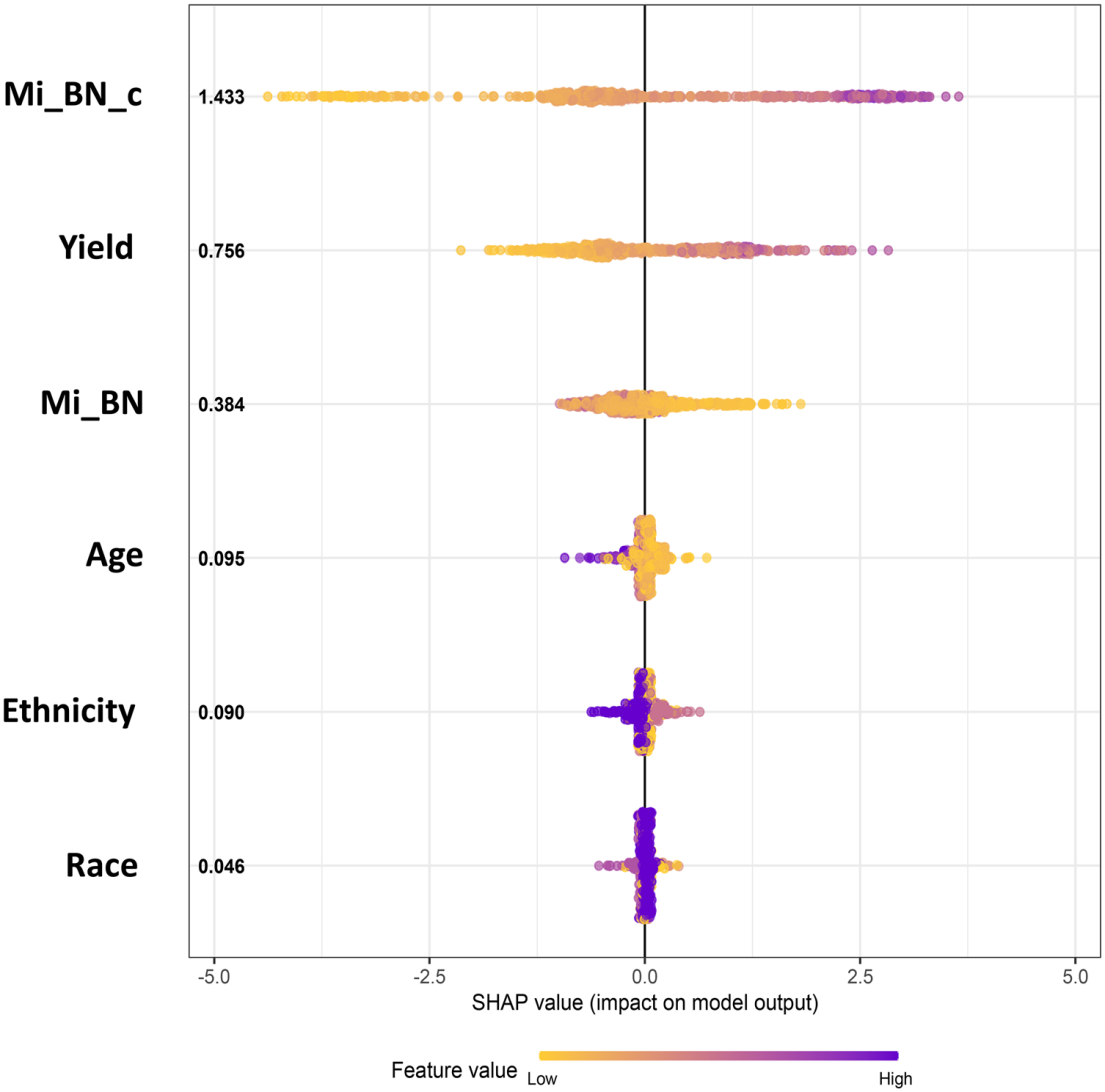
Visualization of how each predictor variable in the random forest model affected the model’s predictions (dose reconstructions). The SHAP metric, explained in the main text, was used to assess the importance of each predictor. Predictor variables (features) are listed on the left side in descending order, based on the mean absolute SHAP value (shown in bold black font). For example, corrected micronuclei yield (Mi_BN_c) was the predictor with the highest mean absolute SHAP value of 1.433 Gy. Negative SHAP values (left side of the figure) represent reductions in reconstructed dose, and positive ones (right side of the figure) represent increases in reconstructed dose. Each circle represents a blood sample (data point). Blue circles represent high feature values, and yellow ones represent low values. For example, high (blue) values of Mi_BN_c were associated with positive SHAP values, i.e. increased reconstructed dose, and low (yellow) values had the opposite effect. Details are discussed in the main text.

**Figure 6:**
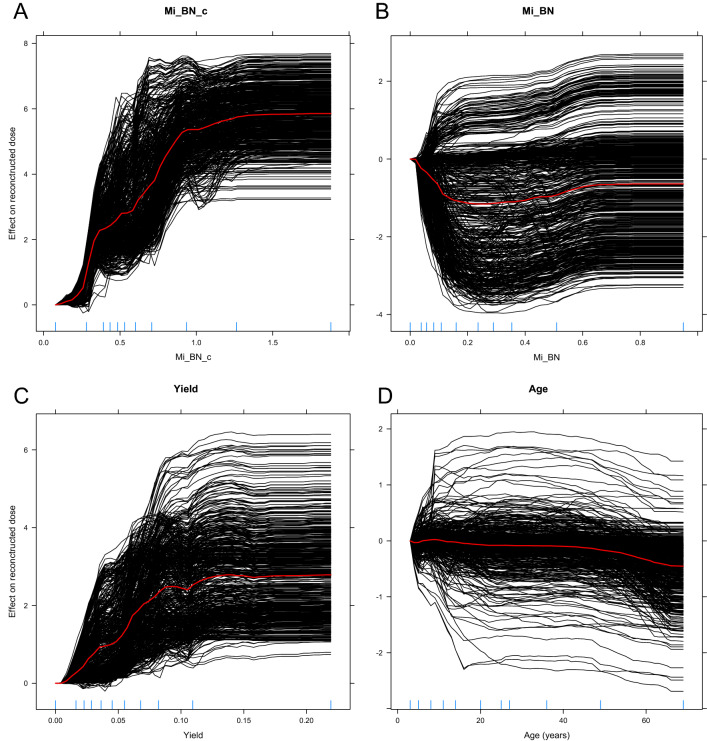
Partial dependence plots, which show the influence of selected predictor variables (**A**–**D**) on reconstructed dose in the random forest model. Each black curve represents an Individual Conditional Expectation (ICE) plot for a given blood sample (data point), which shows how the reconstructed dose for this blood sample changed when the selected predictor variable was changed along the x-axis. Each red curve represents the average of all black curves in each panel.

**Figure 7:**
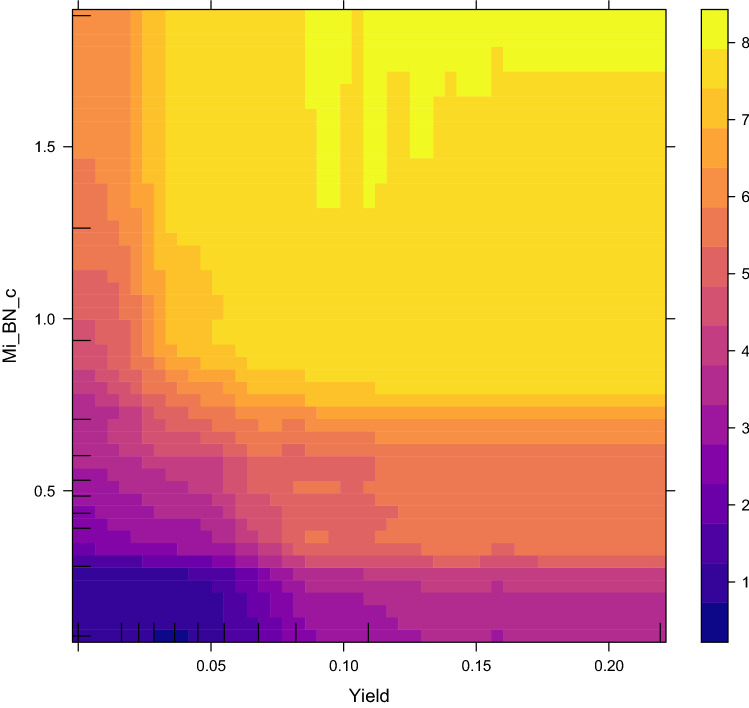
A two-dimensional partial dependence plot, which shows the influence of dicentric chromosomes (Yield) and corrected micronuclei (Mi_BN_c) on reconstructed dose in the random forest model. The plot shows that low values of both Yield and Mn_BN_c are associated with low reconstructed doses (blue), whereas high values of Yield and Mn_BN_c are associated with high reconstructed doses (yellow).

The most important predictor variables in the RF model, assessed by the SHAP metric^42^, were the CBMN and DCA data, followed by age (Fig. 5). SHAP values for the CatBoost model showed very similar patterns (Supplementary Fig. 2). Partial dependence plots, which provide additional details about how the predictions of each model were related to values of features of interest, are displayed in Figs. 6, 7 for RF and Supplementary Fig. 3 for CatBoost. As intuitively expected, the SHAP and partial dependence plots suggest that larger values of corrected micronucleus index (Mi_BN_c) and dicentrics yield (Yield) were associated with higher predicted doses, whereas the corresponding relationship for raw micronucleus index (Mi_BN) was different because of the tendency of this index to decrease at high doses (Fig. 2B).

Older ages were associated with somewhat lower predicted doses, probably as an inverse to the trend for DCA and CBMN yields to increase at older ages (Fig. 3). The effects of Ethnicity and Race variables were generally small and may need to be investigated further in future studies involving even larger data sets.

An alternative calculation, where Dose_rate_category was included in the set of predictor variables for dose reconstruction, was performed with the goal of assessing the magnitude of dose rate effects for the proposed analysis approach. The addition of this extra feature improved the RF performance metrics on testing data only slightly (R^2^ = 0.851, RMSE = 1.140 Gy, MAE = 0.630 Gy). Squared errors were not significantly reduced, compared with the default model without dose rate among the predictors: p-value = 0.157 for a paired Wilcoxon signed rank test in *R.* Therefore, radiation dose reconstructions by the proposed combined method using DCA and CBMN data in an ML framework were not very sensitive to dose rate, even when dose rate was varied over several orders of magnitude. Dose rate effects in this data set were discussed in more detail in our previous publication.

In agreement with our hypothesis that combining DCA with CBMN data would improve dose reconstruction accuracy, removing the Mi_BN variable from the best-performing RF model significantly increased the model’s squared errors on testing data (p-value 3.4 × 10^–8^, using a paired Wilcoxon signed rank test in *R*). R^2^ on testing data was reduced to 0.808, and RMSE and MAE were increased to 1.290 and 0.751 Gy, respectively. Removing both the Mi_BN and Mi-BN_c variables from the model expectedly reduced performance even more: the p-value for a test on squared errors was < 2.2 × 10^–16^, R^2^ was reduced to 0.472, and RMSE and MAE were increased to 2.148 and 1.596 Gy, respectively. Alternatively, retaining Mi_BN and Mi_BN_c variables, but removing Yield from the predictor set, resulted in decreased performance as well, compared with the default model. For the model variant without Yield, R^2^ was reduced to 0.771, and RMSE and MAE were increased to 1.396 and 0.761 Gy, respectively, and the p-value for a test on squared errors, compared with the default model, was 1.1 × 10^–6^. Therefore, using DCA or CBMN data alone resulted in significantly worse dose reconstructions, compared with combining data from both assays.

## Discussion

We hypothesized that combining data from the DCA and CBMN assays using ML approaches can improve the accuracy of radiation dose reconstructions in demographically heterogeneous populations and exposures at different dose rates of ionizing radiation. The rationale for this hypothesis was based on the idea that the DCA and CBMN assays can provide partially complementary (rather than redundant) information, because their dose response shapes and dependences on other factors are not identical (e.g. linear quadratic for DCA and more linear for the “corrected” linearized CBMN index, Fig. 2), and that ML algorithms can extract and utilize this information. To test this hypothesis, we assembled a large data set of ex vivo irradiated blood samples from adult and pediatric blood donors, which was intended to mimic a realistic heterogeneous population of people exposed to a mass-casualty radiological event. We compared the performances of several state-of-the-art ensemble ML methods, e.g. RF, XGBoost, LightGBM, CatBoost, and found that RF and CatBoost models generated the best results based on R^2^, RMSE and MAE metrics. The ensemble tree-based models generally outperformed other algorithms (Table 2). For the RF and CatBoost models, absolute dose reconstruction errors on the testing half of the data were generally well below 1 Gy even though the studied dose range included a high dose of 8 Gy (Fig. 4 and Supplementary Fig. 1). In other words, model performance for dose reconstruction achieved accuracies of < 1 Gy despite the heterogeneity of the subject population (by age, sex, ethnic background, etc.) and the very wide range of investigated dose rates: from 0.08 Gy/day to ≥ 600 Gy/s.

For both the RF and CatBoost models, the most important predictor variables, assessed by the SHAP metric, were the CBMN and DCA data, followed by age (Fig. 5 and Supplementary Fig. 2). Removing either CBMN or DCA data significantly worsened model performance (p-value 3.4 × 10^–8^ for removing Mi_BN and 1.1 × 10^–6^ for removing Yield), compared with using both assays together. These findings demonstrate the promising potential of combining automated CBMN and DCA assays to reconstruct the radiation dose in heterogeneous populations exposed to a mass radiological event. We argue that such a strategy of using ML to integrate the output of different radiation damage assays in the context of high-throughput radiation biodosimetry can help to mitigate the challenges (e.g. different dose rates, radiation qualities) posed by potential improvised nuclear device detonations or other types of malicious or accidental large-scale radiological events in populated areas.

The strengths of the current study include a large and diverse data set, innovative radiation delivery methods which enabled us to investigate both very low and very high dose rates, state of the art ML implementation, and a novel hypothesis. Of course, the study also had limitations. For example, each blood sample was assumed to have the same weight during ML regression analysis, regardless of the number of cells scored for DCA or CBMN assays performed on this sample, although samples with very few cells were removed from the analysis as described in Materials and Methods. The actual dose assigned to each sample was the nominal prescribed dose (e.g. 3 or 8 Gy), rather than a detailed dosimetry estimate on each sample. Radiation type (photons *vs*. electrons) was also not explicitly considered under the assumption that, at the energies used here, both types of exposures represent sparsely ionizing low-LET radiation with similar biological effectiveness. Another reason not to discriminate between photons and electrons in the current context was because electron pulses were used to mimic the very high dose rate prompt photon irradiation after an IND detonation, whereas such dose rates with photons could not be technically achieved in our irradiation facility. Finally, DCA and CBMN assays were scored automatically by a high throughput methodology, which may not be as accurate as manual scoring.

In summary, we proposed a high-throughput radiation biodosimetry approach, which uses ML algorithms to combine the output from automated DCA and CBMN assays. The results showed that combining the assays produced more accurate dose reconstructions, compared with using either assay alone. Although the automated scoring assays are likely to be more error-prone relative to traditional manual scoring, it is advantageous for use in mass-casualty scenarios. High throughput sample preparation, liquid handling and imaging techniques allow for DCA and CBMN assays to be performed on the same sample without excessive use of resources or time. The results of this study demonstrate the promising potential of combining DCA and CBMN assays within the ML framework to reconstruct radiation doses in clinically-relevant radiation exposure scenarios, where the potentially affected population is demographically heterogeneous and radiation dose rates may vary considerably. We also plan future experiments to increase the sample size and diversity in the data set, and potentially to integrate the cytogenetic damage assays with other radiation-responsive biomarker types.

## Supplementary Information

Supplementary Information 1.

Supplementary Information 2.

Supplementary Information 3.

## Data Availability

All datasets analyzed during the current study are available in Supplementary_File_1 online.

## References

[1] Sproull MT, Camphausen KA, Koblentz GD (2017). Biodosimetry: A future tool for medical management of radiological emergencies. Health Secur..

[2] Homer MJ (2016). United states department of health and human services biodosimetry and radiological/nuclear medical countermeasure programs. Radiat. Prot. Dosim..

[3] DiCarlo AL (2021). Scientific research and product development in the United States to address injuries from a radiation public health emergency. J. Radiat. Res..

[4] Rothkamm K (2013). Comparison of established and emerging biodosimetry assays. Radiat. Res..

[5] De Lemos Pinto MMP, Santos NFG, Amaral A (2010). Current status of biodosimetry based on standard cytogenetic methods. Radiat. Environ. Biophys..

[6] Pujol-Canadell M (2020). Cytogenetically-based biodosimetry after high doses of radiation. PLoS ONE.

[7] Repin M, Pampou S, Brenner DJ, Garty G (2020). The use of a centrifuge-free RABiT-II system for high-throughput micronucleus analysis. J. Radiat. Res..

[8] Repin M, Pampou S, Karan C, Brenner DJ, Garty G (2017). RABiT-II: Implementation of a high-throughput micronucleus biodosimetry assay on commercial biotech robotic systems. Radiat. Res..

[9] Royba E (2020). The RABiT-II DCA in the Rhesus Macaque model. Radiat. Res..

[10] Royba E (2019). RABiT-II-DCA: A fully-automated dicentric chromosome assay in multiwell plates. Radiat. Res..

[11] Wang Q (2019). Automated triage radiation biodosimetry: Integrating imaging flow cytometry with high-throughput robotics to perform the cytokinesis-block micronucleus assay. Radiat. Res..

[12] Ryan TL (2019). Optimization and validation of automated dicentric chromosome analysis for radiological/nuclear triage applications. Mutat. Res..

[13] Ryan TL, Pantelias AG, Terzoudi GI, Pantelias GE, Balajee AS (2019). Use of human lymphocyte G0 PCCs to detect intra- and inter-chromosomal aberrations for early radiation biodosimetry and retrospective assessment of radiation-induced effects. PLoS ONE.

[14] Kang CM, Yun HJ, Kim H, Kim CS (2016). Strong correlation among three biodosimetry techniques following exposures to ionizing radiation. Genome Integr..

[15] Lue SW, Repin M, Mahnke R, Brenner DJ (2015). Development of a high-throughput and miniaturized cytokinesis-block micronucleus assay for use as a biological dosimetry population triage tool. Radiat. Res..

[16] Li Y (2019). Radiation dose estimation by completely automated interpretation of the dicentric chromosome assay. Radiat. Prot. Dosim..

[17] Capaccio C (2021). CytoRADx: A high-throughput, standardized biodosimetry diagnostic system based on the cytokinesis-block micronucleus assay. Radiat. Res..

[18] Liu J (2017). Accurate cytogenetic biodosimetry through automated dicentric chromosome curation and metaphase cell selection. F1000Res.

[19] Jang, S. S. *et al.**Automatic Discriminator of Abnormal Chromosomes Using Deep Learning Algorithms.* Report No. 1602.07261v2, (2020).

[20] Shuryak I (2020). A high throughput approach to reconstruct partial-body and neutron radiation exposures on an individual basis. Sci. Rep..

[21] Garty G (2017). Mice and the A-bomb: Irradiation systems for realistic exposure scenarios. Radiat. Res..

[22] Jacobs AR (2020). Role of a high throughput biodosimetry test in treatment prioritization after a nuclear incident. Int. J. Radiat. Biol..

[23] Vral A, Fenech M, Thierens H (2011). The micronucleus assay as a biological dosimeter of in vivo ionising radiation exposure. Mutagenesis.

[24] Wojcik A (2017). The RENEB operational basis: Complement of established biodosimetric assays. Int. J. Radiat. Biol..

[25] Coleman CN, Koerner JF (2016). Biodosimetry: Medicine, science, and systems to support the medical decision-maker following a large scale nuclear or radiation incident. Radiat. Prot. Dosim..

[26] Milner EE (2016). Concepts of operations (CONOPS) for biodosimetry tools employed in operational environments. Health Phys..

[27] Shuryak I (2021). Machine learning methodology for high throughput personalized neutron dose reconstruction in mixed neutron + photon exposures. Sci. Rep..

[28] Pajic J (2015). Inter-individual variability in the response of human peripheral blood lymphocytes to ionizing radiation: Comparison of the dicentric and micronucleus assays. Radiat. Environ. Biophys..

[29] De Amicis A (2014). Dose estimation using dicentric chromosome assay and cytokinesis block micronucleus assay: Comparison between manual and automated scoring in triage mode. Health Phys..

[30] Garty, G., Deoli, N., Obaid, R., Brenner, D. & Kachnic, L. EPD038—the FLASH irradiator at the radiological research accelerator facility. 10.21203/rs.3.rs-1281287/v1 (2022) (**in Press**). 10.1038/s41598-022-19211-7 PMC9780319 36550150

[31] Garty G (2020). VADER: A VAriable Dose-rate External 137Cs irradiatoR for internal emitter and low dose rate studies. Sci. Rep..

[32] Garty, G. *et al.* Sex and dose rate effects in automated cytogenetics. *Radiat. Prot. Dosim.* (2022) (**in Press**). 10.1093/rpd/ncac286 PMC10505938 37721073

[33] Bertucci A, Smilenov LB, Turner HC, Amundson SA, Brenner DJ (2016). In vitro RABiT measurement of dose rate effects on radiation induction of micronuclei in human peripheral blood lymphocytes. Radiat. Environ. Biophys..

[34] R_Core_Team. *R: A Language and Environment for Statistical Computing* (2017).

[35] Kursa MB, Rudnicki WR (2010). Feature selection with the boruta package. J. Stat. Softw..

[36] Breiman L (2001). Random forests. Mach. Learn..

[37] Chen, T. & Guestrin, C. *XGBoost: A Scalable Tree Boosting System*. Report No. 9781450342322, 785–794 (2016).

[38] Ke G (2017). Lightgbm: A highly efficient gradient boosting decision tree. Adv. Neural. Inf. Process. Syst..

[39] Hancock JT, Khoshgoftaar TM (2020). CatBoost for big data: An interdisciplinary review. J. Big Data.

[40] Zhou H, Hastie T (2005). Regularization and variable selection via the elastic net. J. R. Stat. Soc. Ser. B Stat. Methodol..

[41] Awad M, Khanna R (2015). Efficient Learning Machine.

[42] Lundberg, S. M. & Lee, S. I. A Unified Approach to Interpreting Model Predictions. *arXiv* **1705**, 07874.

[43] Bolognesi C (1997). Age-related increase of baseline frequencies of sister chromatid exchanges, chromosome aberrations, and micronuclei in human lymphocytes. Cancer Epidemiol. Biomark. Prev..

[44] Santovito A, Gendusa C (2020). Micronuclei frequency in peripheral blood lymphocytes of healthy subjects living in Turin (North-Italy): Contribution of body mass index, age and sex. Ann. Hum. Biol..

[45] Neri M (2005). Baseline micronuclei frequency in children: Estimates from meta- and pooled analyses. Environ. Health Perspect..

[46] Nersesyan A (2022). Recommendations and quality criteria for micronucleus studies with humans. Mutat. Res. Rev. Mutat. Res..

[47] Stephan G, Pressl S (1999). Chromosomal aberrations in peripheral lymphocytes from healthy subjects as detected in first cell division. Mutat. Res..

[48] Wojda A, Zietkiewicz E, Witt M (2007). Effects of age and gender on micronucleus and chromosome nondisjunction frequencies in centenarians and younger subjects. Mutagenesis.

