# Supplementary material for: A machine learning method for improving the accuracy of radiation biodosimetry by combining data from the dicentric chromosomes and micronucleus assays
Source: Sci Rep. 2022 Dec 6;12:21077. doi: 10.1038/s41598-022-25453-2 (PMC9726929; doi:10.1038/s41598-022-25453-2)
Supplement: Supplementary file 1 — Supplementary Information 1. [file 41598_2022_25453_MOESM1_ESM.docx]

**Supplementary Figure 1.** **Visualization of actual and reconstructed radiation doses by the CatBoost algorithm on the testing half of the data set.** The violin plots for 0, 3 and 8 Gy show the distributions of corresponding reconstructed dose values. The model’s performance metrics were: R^2^ for actual *vs.* reconstructed doses = 0.800, RMSE = 1.304 Gy and MAE = 0.783 Gy. The median reconstructed doses which corresponded to actual doses of 0, 3 and 8 Gy were 0.295, 3.086 and 7.808 Gy, respectively.

**Supplementary Figure 2.** **Visualization of how each predictor variable in the CatBoost model affected the model’s predictions (dose reconstructions).** The SHAP metric is explained in the main text. In panel A, Predictor variables (features) are listed on the left side in descending order, based on the mean absolute SHAP value. Negative SHAP values (left side of the figure) represent reductions in reconstructed dose, and positive ones (right side of the figure) represent increases in reconstructed dose. Each circle represents a blood sample (data point). Red circles represent high feature values, and blue ones represent low values. For example, high (red) values of Mi_BN_c were associated with positive SHAP values, *i.e.* increased reconstructed dose, and low (blue) values had the opposite effect. In panel B, mean absolute SHAP values are shown for each variable, and clustered together in a dendrogram to better visualize which variables provide similar information by clustering together. Details are discussed in the main text. The Race and Ethnicity variables were categorical, and were therefore one hot encoded into separate binary columns for implementation of CatBoost.

**Supplementary Figure 3.** **Partial dependence plots which show the influence of selected predictor variables on reconstructed dose in the CatBoost model.** Each blue curve represents an Individual Conditional Expectation (ICE) plot for a given blood sample (data point), which shows how the reconstructed dose for this blood sample changed when the selected predictor variable was changed along the x-axis. Each yellow dashed curve represents the average of all black curves in each panel.
